# Personalized fluid resuscitation may reduce mortality in septic shock as compared with fixed-volume approach – a systematic review and meta-analysis of randomized controlled trials

**DOI:** 10.1186/s40635-026-00967-5

**Published:** 2026-09-28

**Authors:** Gábor Nagy, Júlia Hollósi, Caner Turan, Dávid Laczkó, Zoltán Sipos, Péter Hegyi, László Zubek, Zsolt Molnár

**Affiliations:** 1 Centre for Translational Medicine, Semmelweis University, Budapest, Hungary; 2 Institute for Translational Medicine, Medical School, University of Pécs, Pécs, Hungary; 3 Institute of Pancreatic Diseases, Semmelweis University, Budapest, Hungary; 4 Department of Anesthesiology and Intensive Therapy, Semmelweis University, Budapest, Hungary; 5 Department of Interventional Radiology, Heart and Vascular Centre, Semmelweis University, Budapest, Hungary; 6 Department of Anesthesiology and Intensive Therapy, Poznan University for Medical Sciences, Poznan, Poland

**Keywords:** Sepsis, Fluid resuscitation, Personalized

## Abstract

**Introduction:**

Immediate fluid resuscitation plays a pivotal role in sepsis management. It remains unclear whether a fixed-volume or personalized strategy is more beneficial. Our aim was to compare effectiveness and safety of personalized vs. fixed-volume resuscitation in septic patients in a systematic review and meta-analysis.

**Methods:**

We conducted a systematic literature search on PubMed, EMBASE and CENTRAL on October 30, 2024. The pre-study protocol was registered with PROSPERO (ID: CRD42024601970). Randomized controlled trials investigated septic patients who received fluid therapy in a personalized way during their initial resuscitation and reported the outcomes of our interest. The meta-analysis was performed using META packages in R with a random-effects model. Risk of bias was assessed using RoB2.

**Results:**

The selection yielded 10 eligible randomized controlled trials (RCTs) with 1,011 patients. Data from 8 RCTs (*n* = 901) revealed significantly reduced odds of 30-day mortality, (OR: 0.62; 95% CI: 0.42–0.93); however, there was no significant difference in the requirement for renal replacement therapy (OR: 0.77; 95% CI: 0.23–2.55), duration of mechanical ventilation (MD: 0.27; 95% CI: -3.09-3.63), vasopressor therapy (MD: -3.70; 95% CI: -32.88-25.48) and length of hospital (MD: -0.87; 95% CI: -4.97-3.25) or intensive care unit stay (MD: 0.44; 95% CI: -1.90-2.77).

**Conclusion:**

Personalized fluid resuscitation, compared with fixed-volume resuscitation, indicated reduced 30-day mortality in septic patients. However, the observed mortality benefit should be interpreted cautiously given the relatively small overall sample size, predominance of small single-center studies, and moderate-to-low certainty of evidence.

**Electronic supplementary material:**

The online version of this article (https://doi.org/10.1186/s40635-026-00967-5) contains supplementary material, which is available to authorized users.

## Introduction

Sepsis is a major healthcare burden, responsible for more than 10 million deaths worldwide [1]. Immediate fluid resuscitation is of paramount importance and may have a significant impact on overall patient outcomes [2].

The current Surviving Sepsis Campaign (SSC) 2026 guideline continues to suggest that adults with sepsis-induced hypoperfusion or septic shock receive at least 30 mL/kg of intravenous crystalloid within the first three hours of resuscitation; however, this is now a weak (conditional) recommendation supported only by low-certainty evidence [3]. Based on this we believe that there is a strong need for further investigating the role of individualized fluid therapy in this patient population. The concurrent 2025 European Society of Intensive Care Medicine (ESICM) guideline on fluid therapy reaches the same position, suggesting up to 30 mL/kg of crystalloid in the initial phase (very low certainty) while endorsing an individualized approach during the optimization phase [4]. This 30 ml/kg approach is based on the pathophysiological rationale that high-volume fluid administration can rapidly correct hypovolemia and improve cardiac output, thereby enhancing oxygen delivery to the tissues [5]. However, there is evidence that acute care providers are unreliable in estimating patient weight, which makes this approach potentially inaccurate [6, 7]. In addition, numerous studies have reported the harmful effects of inappropriate fluid administration, which seriously impairs the function of vital organs such as the kidneys and the heart, and may also lead to insufficient fluid loading or accumulation, causing further adverse events [8, 9].

The optimal fluid resuscitation strategy remains a subject of debate in general.

Numerous studies have investigated the optimal or adequate fluid resuscitation strategy in septic patients, but these studies have often led to contradictory results, despite the variety of approaches to the question [10–13].

In recent years, there has been a strong urge in medicine toward personalized therapy. Resuscitation based on individual physiological needs is considered safer and is widely accepted, but the evidence on its efficacy and safety is limited [13]. For the purposes of this review, we defined personalized fluid resuscitation as any strategy in which the decision to administer or withhold fluid is governed by patient-specific measure of preload/fluid responsiveness.

Considering inconsistency in the literature, we aimed to investigate the effectiveness and safety of personalized fluid resuscitation compared with fixed-volume or other non-personalized approaches in septic patients, by performing a systematic review and meta-analysis.

## Methods

We report our systematic review and meta-analysis based on the recommendations of the PRISMA 2020 guidelines [14], while we followed the Cochrane Handbook [15]. The PRISMA checklist is included in the Supplementary Material. The protocol of the study was registered with PROSPERO (registration number CRD42024601970), and we adhered to it.

### Eligibility criteria

Only randomized controlled trials (RCTs) published in peer-reviewed scientific journals were included in this study. We report the study framework and eligibility criteria according to the PICO method, where population (P): adult septic patients of both sexes (aged 18 years or older); intervention (I): personalized fluid resuscitation guided by assessment of fluid responsiveness of patients; control (C): patients resuscitated with fixed-volume of fluid in a non-personalized way, without the assessment of fluid responsiveness. Main outcome (O): 30-day mortality and secondary outcomes were organ-support-free days, length of ICU and hospital stay.

Exclusion criteria included study designs other than RCTs, animal studies, traumatic and pregnant patients and patients without sepsis. No studies with overlapping populations were included.

### Search strategy

Our systematic search was conducted on October 6, 2024 on MEDLINE via PubMed, Embase, and the Cochrane Central Register of Controlled Trials (CENTRAL) databases without filters and restrictions on publication date, language, or article type. A repeated search was performed on July 14, 2026, to detect any new relevant publications.

The following search key was used in the systematic search: (”sepsis” OR ”septic”) AND ((personali* OR individual* OR standard* OR “usual” OR protocol* OR guideline* OR (“goal” AND “directed”)) AND (“fluid” AND (“therapy” OR resuscitat* OR replac*))). A modified search key was used for the search on Embase and CENTRAL (sepsis OR septic) AND ((personali* OR individual* OR standard* OR usual OR protocol* OR guideline* OR (goal AND directed)) AND (fluid AND (therapy OR resuscitat* OR replac*))). References in the included articles were checked for additional eligible studies; however, it did not result in the inclusion of further articles.

### Selection process

After searching various databases, we exported the results to an Endnote file. Duplicates were detected and removed using both automatic and manual searches. The selection was performed by two – blinded and – independent review authors (GN as review author 1 and JH as review author 2). The two reviewers assessed the results for inclusion, first by screening title sand abstracts, then by reviewing full texts. As agreed, any conflicts were resolved by a third independent investigator (DL). Cohen’s Kappa was calculated two times to evaluate inter-reviewer agreement: after title-and-abstract selection and after full-text selection, with K = 0.88 and K = 0.87, respectively. The degree of agreement had to reach the level of 0.8.

### Data collection process

Data were independently collected by two authors (GN and JH) from eligible articles. Disagreements were resolved through discussion between the authors. We created a standardized Excel data extraction form (Microsoft Excel, Microsoft Corporation) based on the consensus of methodological and clinical experts. The following data were extracted: study characteristics: first author, year of publication, study design, study population (number, age, and sex of participants), method of personalizing fluid therapy and definition of sepsis used across the studies. Study period, study country, and number of centers are detailed in Supplementary Table 1; 30-day mortality; organ-support-free days including duration of vasopressor therapy, duration of mechanical ventilation, requirement for renal replacement therapy (RRT), vasopressor- and mechanical ventilation-free days; and length of ICU stay in addition to the time spent in the hospital.

### Study risk of bias assessment and the certainty of evidence assessment

Two authors (GN, JH) independently performed the risk of bias assessment, according to the recommendations of the Cochrane Handbook [15] using the RoB 2 tool [16]. Disagreements were resolved through discussion and consensus between the authors. The risk of bias assessed five distinct domains, including the randomization process, deviations from intended intervention, missing outcome data, outcome measurement, selection of reported outcomes, and overall bias. Due to the nature of the intervention blinding of clinicians to treatment allocation was inherently impossible in all included trials.

The certainty of evidence was assessed using the GRADE approach, following the GRADE Handbook [17], with analyses performed using the online software GRADE Pro GDT version 20 [18].

### Synthesis methods

Meta-analysis was performed for outcomes for which data were reported in at least three separates, included studies. Both qualitative and quantitative syntheses of the data were performed, and a random-effects model was used for the analysis. For dichotomous outcomes, odds ratios (ORs) were used, and for continuous outcomes, mean differences (MDs) were calculated to measure the effect, each with a 95% confidence interval (CI). We used the META (Schwarzer 2022, v5.2.0) and DMETAR (Cuijpers, Furukawa, and Ebert 2022, v0.0.9000) packages for calculations and plots. For studies that did not report the mean and standard deviation, estimations were made using the available medians, quartiles, minimum, and maximum values using the methods of Luo [19] and Shi [20]. Forest plots were used to visualize and summarize our results. I² and χ² tests were used to assess statistical heterogeneity with a statistical significance threshold of *p*-value < 0.1. The thresholds for I² were considered according to the recommendations of the Cochrane Handbook: 0–25% as low, 25–50% as moderate and 50–100% as considerable heterogeneity. Due to the low number of eligible studies, publication bias could not be assessed by performing Egger’s test.

## Results

### Search and selection

**Fig. 1:**
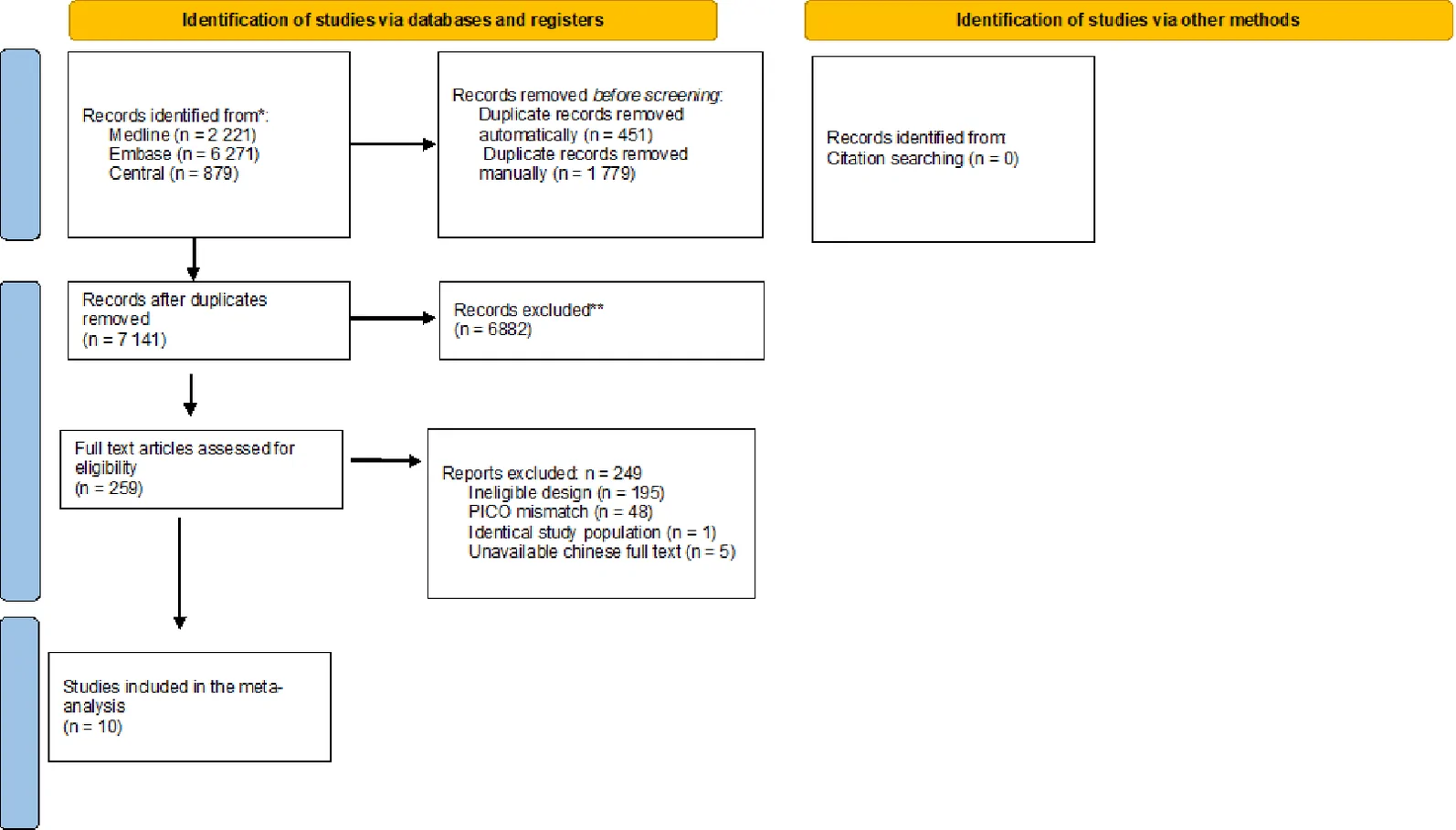
PRISMA 2020 flowchart illustrating the systematic search and study selection process

During our systematic search, a total of 9,371 studies were identified. The selection process is detailed in the PRISMA flowchart, as presented in Fig. 1. Overall, 10 randomized controlled trials [21–30] were included in our study, the updated systematic search and citation chasing yielded no additional eligible trials (Supplementary Fig. 1).

### Basic characteristics of included studies and patients

Altogether, we analyzed data from 1,011 patients, of whom 542 received personalized treatment in the intervention group, and 469 were in the control group. Table 1. details baseline characteristics, clinical data, and intervention summaries of the included articles. In the study by Afandy et al. [21], the allocation sequence was 1:1:1. Two interventional arms were applied during randomization (30 participants were assigned to the cardiometry group and 30 patients in echocardiography group), and they were merged based on expert statistical considerations.

**Table 1:** Basic characteristics of included studies

Author and publication date	Sepsis definition	Patient distribution		Age, years		Sex, Female % of total	
		Intervention	Control	Intervention	Control	Intervention	Control
Afandy et al. 2020 [21]	SEPSIS-3	60	30	38.5 ± 3.3^a^	39.4 ± 3.7^a^	43.3	46.7
Alhabashy et al. 2021 [22]	SEPSIS-2	42	45	42.3 ± 8.96^a^	44.44 ± 7.65^a^	42.9	48.9
Chen et al. 2015 [23]	unspecified	41	41	60 (50,68)^b^	58 (45,65)^b^	51.2	48.8
Douglas et al. 2020 [24]	SEPSIS-1	83	41	61.8 ± 16.9^a^	62.7 ± 15^a^	61.4	31.7
Elsayed et al. 2021 [25]	SEPSIS-3	50	50	60.56 ± 15.12^a^	58.28 ± 17.45^a^	44	48
Ismail et al. 2019 [26]	SEPSIS-3	40	40	56^c^	58^c^	45	57.5
Kuan et al. 2016 [27]	unspecified	61	61	66.6 ± 14.9^a^	64.3 ± 14.6^a^	49.2	34.4
Lanspa et al. 2018 [28]	SEPSIS-2	15	15	69 (61,77)^b^	64 (49,75)^b^	53	47
Li et al. 2021 [29]	unspecified	49	45	54.4 ± 15.2^a^	56.7 ± 11.0^a^	51	37.7
Musikatavorn et al. 2021 [30]	SEPSIS-3	101	101	65.3 ± 20.1^a^	63.7 ± 16.8^a^	47.5	37.6

a = mean±standard deviation; b = median (interquartile range); c = median

TTE: transthoracic echocardiogram; IVC: inferior vena cava; LVOT: left ventricular outflow tract; VTI: velocity time integral; PLR: passive leg raising; SV: stroke volume; PPV: pulse pressure variation; CO: cardiac output; IVC-CI: inferior vena cava collapsibility index

#### Characteristics of Included Populations and Interventions

Baseline illness severity and resuscitation-related parameters were reported inconsistently across the ten included trials (Supplementary Table 2). Illness severity was characterized by the SOFA score in six trials and by the APACHE II score in seven; where reported, SOFA scores ranged from approximately 3 to 13 and APACHE II scores from approximately 21 to 33. Baseline lactate, available in eight trials, was consistently elevated (means or medians of approximately 3 to 6 mmol/L). Within each trial, severity scores and lactate concentrations were generally comparable between the randomized groups, supporting adequate baseline balance. Vasopressor dosing was reported in only four trials and in heterogeneous units and metrics (e.g., mean baseline dose in µg/kg/min versus maximum dose in µg/min), precluding direct comparison, and two trials reported point estimates without any measure of dispersion, further limiting quantitative synthesis of baseline characteristics.

The reported source of sepsis varied between trials, both in its distribution and in the manner in which it was categorized (Supplementary Table 3). The respiratory tract (pneumonia) was the most frequently identified focus in the majority of trials that reported this information, typically accounting for approximately one-third to one-half of cases. Three trials [23, 27, 30] did not report the source of infection, and one trial [24] classified infections by pathogen type rather than by anatomical site.

All trials used crystalloids for resuscitation; two specified 0.9% saline (with or without Ringer’s lactate) and the remainder reported “crystalloid” without further detail. Personalization of fluid therapy was achieved predominantly through dynamic, point-of-care ultrasound and echocardiographic assessment of fluid responsiveness (Table 2). The most common approach combined a passive leg raise with a stroke-volume or flow surrogate (stroke volume, stroke-volume index, or the velocity–time integral of the carotid artery or left-ventricular outflow tract); inferior vena cava diameter and collapsibility and focused echocardiographic indices of cardiac function were also widely used, while one trial relied on lung-ultrasound B-lines. Response thresholds differed substantially between trials.

**Table 2:** Precision fluid therapy: Selection and individualization

Author and publication date	Fluid type	Way of personalization	Target
Afandy et al. 2020 [21]	crystalloid	Fluid resuscitation driven by cardiometry and echocardiography	LVEF > 40% SVV with unspecified target value
Alhabashy et al. 2021 [22]	crystalloid	Fluid resuscitation driven by TTE + IVC and LVOT VTI	IVC diameter < 2 cm and IVC-CI > 50% increase in LVOT VTI > 12%
Chen et al. 2015 [23]	Normal saline or RL	Fluid resuscitation driven by PLR + IVC distension index, SV index and PPV	PPR decreased to < 13%, IVCDI decreased to < 18%, SVI increased by > 10 (at least two of these parameters had to be met)
Douglas et al. 2020 [24]	crystalloid	Fluid resuscitation driven by PLR + SV (contractility)	SV increased by at least 10%
Elsayed et al. 2021 [25]	crystalloid	Fluid resuscitation driven by PLR + Carotid VTI	carotid VTI increased > 10% with PLR
Ismail et al. 2019 [26]	crystalloid	Fluid resuscitation driven by lung ultrasound applied on four intercostal space: the 3rd and the 4th intercostal space on the right and the 6th and 7th intercostal space on the left between the midclavicular and parasternal line.	less than 4 B-lines/intercostal space
Kuan et al. 2016 [27]	crystalloid	Fluid resuscitation driven by PLR + non-invasive CO monitor	SVI increase > 10% with PLR
Lanspa et al. 2018 [28]	crystalloid	Fluid resuscitation driven by IVC-CI and myocardial function via TTE	IVC-CI > 50% LVEF > 45% RV FAC> 35% TAPSE > 16 mm
Li et al. 2021 [29]	crystalloid	Fluid resuscitation driven by cardiopulmonary ultrasound (dilated RV size, severely or moderately impaired LV systolic function, EVLW) and IVC diameter ≥ 20 mm	if none of these was applied – 30 ml/kg fluid was administered if any of these was applied – fluid resuscitation was cautiously performed if 2 of these were applied – fluid resuscitation was dispensed and vasopressor support was enhanced
Musikatavorn et al. 2021 [30]	Normal saline	Fluid resuscitation driven by IVC- CI and diameter	IVC diameter during inspiration and collapsibility index (goal: > 40)%

LVEF: left ventrucular ejection fraction, SVV: stroke volume variation, TTE: transthoracic echocardiography, IVC: inferior vena cava, LVOT VTI: left ventricular outflow tract velocity time integral, IVC-CI: inferior vena cava collapsibility index, PLR: passive leg raising, PPV: pulse pressure variation, PPR: pulse pressure ratio, IVCDI: inferiosr vena cava distension index, SVI: stroke volume index, CO: cardiac output, RV FAC: Right ventricular fractional area change, TAPSE: Tricuspid annular plane systolic excursion, EVLW: extravascular lung water

### Primary outcome

#### 30-day mortality

Eight [21–27, 29, 30] (*n* = 901) out of ten eligible articles in this study reported 28-day or 30-day mortality as an outcome. To maximize statistical power, 28-day and 30-day mortality rates were pooled together under this single endpoint.

**Fig. 2:**
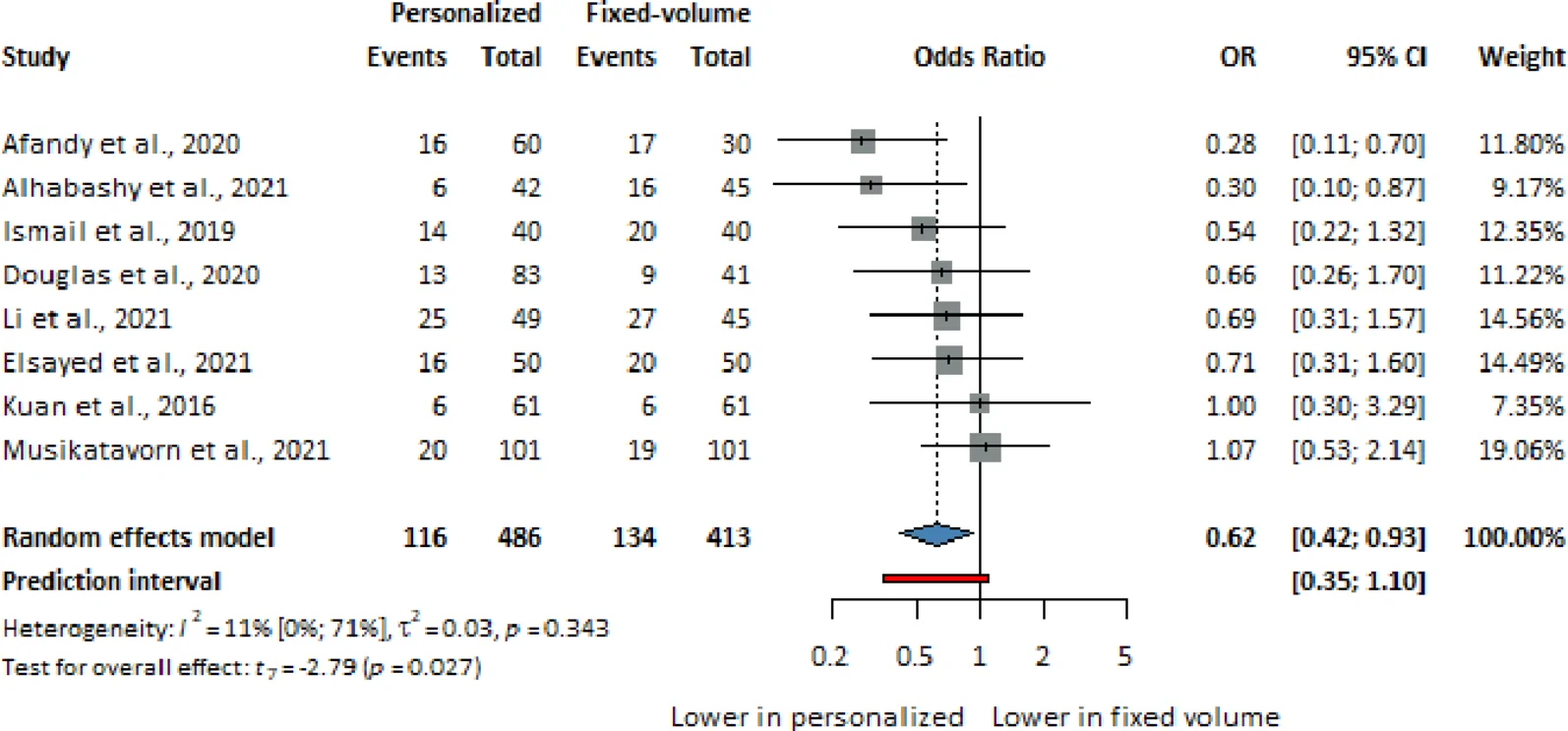
Forest plot illustrating the odds of 30-day mortality

In this analysis, 486 patients were in the intervention group who received fluid resuscitation in a personalized way, and 415 were treated with fixed-volume fluid administration (Fig. 2). Our results showed a statistically significant lower 30-day mortality in the personalized group (OR: 0.62; 95% CI: 0.42–0.93, *p* = 0.027). Moderate heterogeneity was observed [I^2^ = 22% (0%;71%), *p* = 0.343] for this outcome.

To evaluate the robustness of the pooled 30-day mortality estimate, we performed a leave-one-out sensitivity analysis by sequentially omitting each of the eight contributing trials and re-estimating the random-effects model (Supplementary Fig. 2). The pooled effect was directionally stable across all iterations; re-calculated odds ratios consistently favoured personalized resuscitation, ranging from 0.55 (95% CI, 0.37–0.82) to 0.70 (95% CI, 0.49–1.00). Furthermore, every leave-one-out estimate fell well within the confidence interval of the primary analysis. Between-study heterogeneity remained low to negligible throughout (I2: 0–24%), dropping to 0% when *Musikatavorn et al. (2021)*, *Alhabashy et al. (2021)*, or *Afandy et al. (2020)* was excluded.

However, the strict statistical significance of the primary finding was contingent upon a few influential trials. The mortality benefit was attenuated to non-significance upon the omission of *Alhabashy et al. (2021)* (OR, 0.67; 95% CI, 0.45–1.01), *Ismail et al. (2019)* (OR, 0.63; 95% CI, 0.39–1.02), or *Afandy et al. (2020)* (OR, 0.70; 95% CI, 0.49–1.00), whereas it retained significance under the remaining five omissions. Overall, while the direction and clinical magnitude of the 30-day mortality effect are robust to the removal of any single trial, its statistical significance relies on a subset of the included studies.

### Secondary outcomes

#### Length of ICU stay

Across seven studies [21–24, 27, 29] (*n* = 819), we observed no statistically significant difference in the number of days spent in the ICU between patients receiving personalized therapy (*n* = 447) and those receiving fixed-volume fluid administration (Supplementary Fig. 3 A) (*n* = 372) (MD: 0.44; 95% CI: −1.90-2.77, *p* = 0.663). A considerable degree of heterogeneity was observed for this endpoint [I^2^ = 80% (59%;90%), *p* < 0.001].

#### Length of hospital stay

A total of four articles [21, 22, 24, 27] (*n* = 423) reported this outcome between the two groups of patients (*n* = 246 vs. *n* = 177). Our analysis found no statistically significant difference between the groups (Supplementary Fig. 3B) (MD: −0.87; 95% CI: −4.97-3.23, *p* = 0.547), with considerable heterogeneity [I^2^ = 84% (61%;94%), *p* < 0.001].

#### Renal replacement therapy

A total of four studies [23, 24, 29, 30] reported the requirement for renal replacement therapy (*n* = 502) (Supplementary Fig. 4 A). We found no statistically significant difference between patients receiving personalized fluid therapy (*n* = 275) and those receiving fixed-volume fluid therapy (*n* = 227).

(OR: 0.77; 95% CI: 0.23–2.55, *p* = 0.53) with moderate heterogeneity [I^2^ = 46% (0%;82%), *p* = 0.137].

#### Mechanical ventilation

Five studies [21–23, 25, 29] reported duration of mechanical ventilation as an outcome (*n* = 453) (Supplementary Fig. 4B) with considerable heterogeneity [I^2^ = 86% (69%;94%), *p* < 0.001]. We found no statistically significant difference (MD: 0.27; 95% CI: −3.09-3.63, *p* = 0.835) for this outcome.

#### Circulatory support

Duration of vasopressor therapy was reported in five studies [21–23, 25, 29]. No statistically significant difference was observed in the duration of circulatory support between the personalized and fixed-volume groups (MD: −3.70; 95% CI: −32.88-25.48, *p* = 0.743) (Supplementary Fig. 4 C) and there was considerable heterogeneity [I^2^ = 97% (96%;98%), *p* < 0.001].

### Findings on fluid administration

Administered fluid volumes and fluid balance data reported in the included trials are summarized in Table 3.

**Table 3:** Fluid-related outcomes in the included trials: Study fluid volume, total fluid intake, and cumulative fluid balance by treatment arm and time point

Author	Comparison of fluid therapy related data				Comment
		Intervention	Control	*p*-value	
Elsayed et al. 2021 [25]	Administered fluid by the the end of 6 h	1750^a^	3000^a^	< 0.001	Significantly lower amount of fluid was administered in the treatment group with non-significant mortality benefit
Musikatavorn et al. 2021 [30]	Cumulative fluid used at 6 h	19,006(1500–2570)	2600(2300–3220)	< 0.001	Significantly lower volume of fluid was used in treatment group without beneficial effect on mortality
	Cumulative fluid used at 24 h	4080(2990–5255)	4800(3810–6410)	< 0.01	
Ismail et al. 2019 [26]	Daily balance on the first day	900^a^	1850^a^	< 0.001	Significantly lower fluid balance in treatment group on the first day with non-significant mortality benefit
Douglas et al. 2020 [24]	Fluid balance at 72 h	530(−840-2530)	1220(−30-3730)	0.021	Significantly lower 3rd day fluid balance and total amount of fluid in treatment group with non-significant mortality benefit
	Total fluid at 72 h	3354 ± 2179	4721 ± 3319	N/A**	
Alhabashy et al. 2021 [22]	Fluids in the first 24 h	2564 ± 927	3635 ± 973	< 0.01	Significantly lower volume of fluid was administered in treatment group with significant mortality benefit
Afandy et al. 2020 [21]	Cumulative volume of treatment fluids	2545(2050–3490) and 2470(2000–3400)	3615(2456–4800)	< 0.01	Significantly lower volume of fluid was administered in treatment group with significant mortality benefit
Li et al. 2021 [29]	Fluid balance within the initial 6 h	2092 ± 1453	1508 ± 1211	0.04	Significantly lower fluid balance in the control group without significant difference administered fluid volume with non-significant mortality benefit
	Volume of fluid intake	2568.2 ± 1142.6	2111 ± 1115	N/A*	
Chen et al. 2015 [23]	Net balance at 72 h	1950^a^	3124^a^	0.2	No significant difference between groups
	Net balance at the end of 5th day	2640^a^	3610^a^	0.4	
	Study fluid at 72 h	4410^a^	4100^a^	0.47	
	Study fluid at 5th day	6200^a^	8700^a^	0.26	
Lanspa et al. 2018 [28]	24 h fluid administration	6000(470–8500)	6400(4800–9600)	0.63	No significant difference between groups
Kuan et al. 2016 [27]	Cumulative volume of fluid at 1 h	1000(500–1000)	500(250–1000)	N/A*	No significant difference between groups without beneficial effect on mortality
	Cumulative volume of fluid at 2 h	1500(1100–2000)	1100(600–2100)	N/A*	
	Cumulative volume of fluid at 3 h	2100(1600–2550)	1600(1000–2700)	N/A*	
	Cumulative volume of fluid at 24 h	3550(2850–4800)	3400(2100–5600)	N/A*	
	Cumulative volume of fluid at 72 h	5100(3500–6800)	5400(3250–8200)	N/A*	

^a^ median * reported as non-significant without exact p-value ** reported as significant without exact p-value

### Risk of bias assessment

**Fig. 3:**
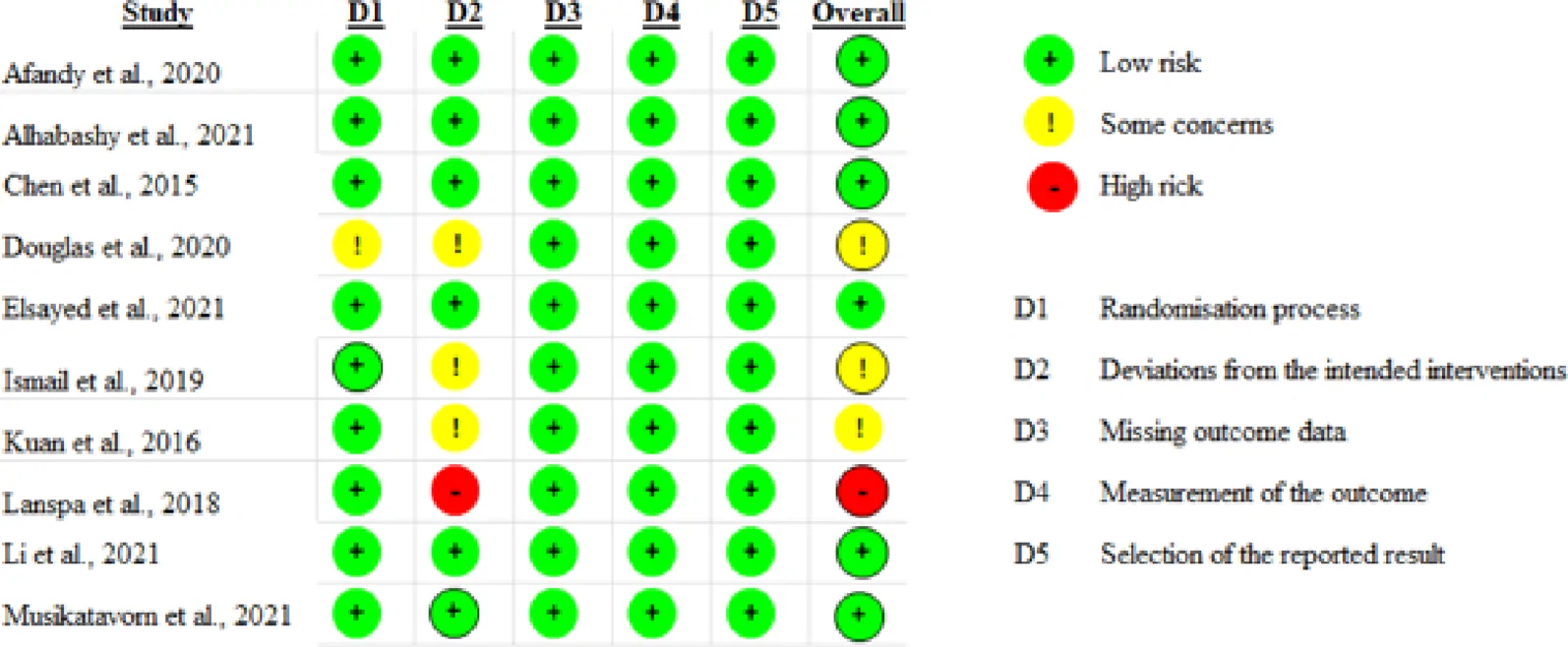
Risk of bias assessment

The results of the risk of bias assessment are presented in Fig. 3, which presents the results of the assessment of all outcomes.

Overall, most studies were adequately randomized, and none exhibited concerns related to missing outcomes. A moderate risk of bias was identified due to unequal gender distribution, or deviations from the intended treatment; however, these did not seriously affect the outcome. Only one article was assessed as having high overall risk of bias due to serious deviations from the intended intervention.

### Heterogeneity

Levels of heterogeneity are interpreted according to the Cochrane Handbook [15] using t^2^ and I^2^ test statistics. Moderate heterogeneity was observed in the analysis of 30-day mortality and requirement for renal replacement therapy; furthermore, considerable heterogeneity was observed across the other studies. These findings may be explained by the low number of studies included and the fact that participants had different underlying conditions and baseline characteristics. Another potential reason is the use of different methods and tools to assess fluid responsiveness as a guide for treatment. In addition, differences in the definition of sepsis and septic shock in the studies may also have contributed to the observed level of heterogeneity. Each secondary outcome’s leave-one-out analysis and the impact on statistical heterogeneity are provided in the supplementary material. (Supplementary Fig. 6A-E and 7 A-E)

### Certainty of evidence assessment

Studies were also evaluated for certainty of evidence using the GRADE assessment framework. Detailed results of the GRADE assessment for meta-analyzed outcomes are presented in Table 4. Overall, the certainty of the evidence ranged from very low to moderate.

**Table 4:** Summary of findings and certainty of the evidence (GRADE) for personalized compared with fixed-volume fluid resuscitation in adults with septic shock: Results of random-effects meta-analyses of 10 randomised controlled trials

Certainty assessment							№ of patients		Effect		Certainty	Importance
№ of studies	Study design	Risk of bias	Inconsistency	Indirectness	Imprecision	Other considerations	Personalized fluid therapy	Fixed-volume fluid therapy	Relative (95% CI)	Absolute (95% CI)		
**30-day mortality (follow-up: 30 days; assessed with: Odds ratio)**												
8	RCT	Serious	Not serious	Not serious	Not serious	None	116/486 (23.9%)	134/413 (32.4%)	**OR 0.62** (0.42 to 0.93)	**95 fewer per 1000**	⨁⨁⨁◯ Moderate	CRITICAL
**Length of hospital stay (assessed with: mean)**												
4	RCT	Serious	Serious	Not serious	Not serious	None	246	177	-	MD **0.87 days lower** (−4.97-3.23)	⨁⨁◯◯ Low	IMPORTANT
**Requirement for renal replacement therapy (assessed with: Odds ratio)**												
4	RCT	Not serious	Serious	Not serious	Not serious	None	53/275 (19.3%)	57/227 (25.1%)	**OR 0.77** (0.23 to 2.55)	**46 fewer per 1000**	⨁⨁⨁◯ Moderate	IMPORTANT
**Duration of mechanical ventilation (assessed with: mean)**												
5	RCT	Not serious	Serious	Not serious	Not serious	None	243	210	-	MD **0.27 day higher** (3.09–3.63)	⨁⨁⨁◯ Moderate	IMPORTANT
**Mechanical ventilation free days (assessed with: mean)**												
3	RCT	Serious	Not serious	Not serious	Not serious	None	98	101	-	MD **0 days** (−1.05 −1.04)	⨁⨁⨁◯ Moderate	IMPORTANT
**Duration of vasopressor therapy (assessed with: mean)**												
5	RCT	Not serious	Very serious	Not serious	Not serious	None	243	210	-	MD **3.7 days lower** (−32.88-25.48)	⨁⨁◯◯ Low	IMPORTANT
**Length of Intensive Care Unit stay (assessed with: mean)**												
7	RCT	Not serious	Very serious	Not serious	Serious	None	447	372	-	MD **0.44 days higher** (−1.9-2.77)	⨁◯◯◯ Very low	IMPORTANT

CI: confidence interval; MD: mean difference; OR: odds ratio

## Discussion

To our knowledge, this study is the first comprehensive systematic review and meta-analysis on the safety and effectiveness of personalized fluid resuscitation versus fixed-volume resuscitation in septic patients. Our results revealed that personalized fluid resuscitation based on the physiological needs and individual fluid responsiveness of patients was associated with a lower 30-day mortality, however while the direction and clinical magnitude of this benefit remained robust during sensitivity analysis, its strict statistical significance proved sensitive to the removal of individual trials. Consequently, while the overall trend consistently favours the personalized approach, the fragility of the statistical signal indicates these results should be interpreted with caution.

Earlier versions of SSC guidelines advocated a quantitative resuscitation protocol – including aggressive fluid resuscitation – based on the early goal-directed therapy study published by Rivers et al. in 2001 [31]. This was followed by three additional clinical trials (PROCESS, ARISE and PROMISE) [32–34], the results of which called the earlier recommendation into question. In these trials, clinicians administered an average of 30 ml/kg of fluid prior to randomization, a practice appearing in the guidelines since. Successive SSC guidelines reflect a progressive de-emphasis of the fixed 30 mL/kg target: a strong recommendation in 2012, retained in 2016 and 2021, and subsequently rendered a weak (conditional) recommendation on low-certainty evidence in 2025 ESICM and the current 2026 guideline, which explicitly endorse individualized titration and frequent reassessment [3, 35].

Over the past decade, several studies on fluid therapy have addressed the question of whether a liberal or restrictive approach to fluid therapy improves outcomes, although the results are still controversial: the CLOVERS study found no significant difference between the groups [36], while a retrospective observational study by Jiang et al. showed a significantly improved mortality in the restrictive group [37]. In addition, Rivers et al. observed a significant reduction in mortality with liberal fluid therapy [31]. Although a trend toward lower mortality was observed in the restrictive group in six studies included in our analysis [21, 22, 24–27], this difference reached statistical significance in only two of them [21, 22]. In one of our recent meta-analysis we also found that perfusion guided management, which can also be considered a personalized approach, led to significantly better outcomes with less fluid administration in the treatment arm [38]. This raises doubts as to whether the right question during fluid resuscitation is whether we should be restrictive or liberal, but rather how to administer the amount of fluid that the patient really requires.

In 2021, Vincent et al. published 20 recommendations on how to individualize early resuscitation in septic patients [13]. Our results provide further support to this expert opinion, as we found that the use of dynamic parameters to guide fluid therapy resulted in a beneficial effect on mortality [21, 22, 24, 25, 27, 29]. Although we were unable to meta-analyze the amount of fluid administered and its effect on outcomes, the extracted results indicate that patients in the individualized group more frequently received less fluid and had better outcomes [20, 21, 23–26], but no firm conclusions could be drawn.

Our findings have be interpreted in light of the most recent evidence, the ANDROMEDA-SHOCK-2 trial Although its hierarchical composite primary outcome favored the personalized strategy and the intervention reduced early resuscitation fluid and increased vital-support-free days, 28-day mortality was virtually identical between groups [39], which tempered the mortality signal in our pooled analysis. Our results are nonetheless concordant in direction with two contemporary researches. In 2025 Wang et al. restricting eligibility to dynamic fluid-responsiveness strategies across 9 RCTs, reported that such strategies probably reduce 28-day mortality (RR 0.61, 95% CI 0.42–0.90; moderate certainty) and reduce acute kidney injury and day-3 fluid balance) [40]. The divergence in certainty is instructive: their narrower, more homogeneous intervention yielded a more robust estimate, whereas our broader definition, which also admits perfusion-endpoint and fluid-minimization strategies, together with the inclusion of small single-center trials, renders our signal more fragile. The network meta-analysis by Yumoto et al. in 2023. found that lactate- or capillary refill time–guided resuscitation may reduce short-term mortality [41] its network framework is better suited than pairwise pooling to rank specific monitoring and target modalities, and its perfusion-targeted signal coheres with the organ-support benefit seen in ANDROMEDA-SHOCK-2.

We also found some important differences in the various methods and measurements used to assess fluid responsiveness during the personalized fluid therapy. IVC-based therapy proved to be less accurate, while more complex methods based on the combined measurement of multiple hemodynamic parameters provided a more coherent picture, consistently reducing inappropriate fluid administration, which can affect mortality in the long term [22, 24].

Neverthelelss, the concept of “personalized fluid therapy” remains broad and inconsistently defined across the included trials. This raises concern regarding whether the pooled interventions truly represent a comparable therapeutic strategy that should be taken into account when interpreting our results.

The observed reduction in pooled mortality in the absence of consistent improvement in secondary organ-support outcomes warrants careful interpretation. This apparent dissociation does not undermine our primary finding, rather reflects the complex interplay of statistical heterogeneity, differential trial design, and the mechanistic pathways through which personalised resuscitation exerts its effects, or underpowered trials [23, 28, 29]. The predominant mechanism through which excess fluid volume causes early mortality is temporally decoupled from the processes determining organ support duration in surviving patients. Accordingly, mortality reduction and improvements in secondary outcomes need not be expected to co-occur within the same analysis, particularly when the included trials are heterogeneous in experimental separation, sample size, and co-intervention protocols. This is also underscored by the ADROMEDA-SHOCK-2 trial, which found that there was significant benefit in the composite outcome in the treatment group without a significant effect on mortality. Our findings should be interpreted as hypothesis-generating, supporting the design of adequately powered multi-centre trials with pre-specified, standardised secondary outcome definitions.

Furthermore, it is important to note that outcomes, such as length of ICU stay and duration of organ support can be misleading, especially when early deaths in the ICU are compared with survivors requiring prolonged treatment and care. Therefore, these outcomes should be reported as “organ support-free days” in future clinical trials [42], which was not the case in the studies included in the analysis.

### Strengths and limitations

In terms of the strengths of our analysis, we included only randomized controlled trials to generate the highest level of evidence, followed our protocol that was registered in advance.

Our study was limited by substantial clinical and methodological heterogeneity across the included trials. Second, the observed mortality benefit was based on small, single-center studies. Furthermore, it is also important to note that statistical significance was lost when any one of three key trials was omitted by applying the leave-one-out analysis. Third, prevented us from performing subgroup analyses and relatively small number of patients, along with moderate to high heterogeneity in assessment and treatment, may limit the generalizability of our findings. Fourth, unified and consequent definition of sepsis and septic shock also varied across the studies or was even missing. Finally, due to the small number of studies, publication bias could only be assessed by Funnel plots (Supplemetary Fig. 5A-F).

### Implications for practice and research

The translation of scientific results into patient care is of outmost importance [43, 44]. Our results strongly support the prioritization of personalized strategies in fluid resuscitation in septic shock (Supplementary Fig. 8) [45].

We recommend a reorientation of research questions in this field: rather than investigating whether restrictive or liberal fluid therapy is more superior, the focus should be on identifying the best approach to personalizing fluid therapy [46, 47].

## Conclusion

This meta-analysis provides evidence that personalized fluid therapy during initial resuscitation of septic patients may be associated with a significant improvement in survival compared with fixed-volume treatment. However, the observed mortality benefit should be interpreted cautiously given the relatively small overall sample size, predominance of small single-center studies, and moderate-to-low certainty of evidence. Adequately powered, multicenter randomized controlled trials with blinded outcome assessment, appropriate endpoints (e.g., organ support-free days) and a standardized definition of individualized resuscitation are needed to determine the optimal way of personalizing initial resuscitation in sepsis.

## Electronic supplementary material

Below is the link to the electronic supplementary material.

Supplementary material 1 (DOCX 19,190 kb)

## Data Availability

The datasets used in this study can be found in the full-text articles included in the systematic review and meta-analysis.

## Abbreviations

OR: Odds Ratio
CI: Confidence Interval
MD: Mean Difference
RCT: Randomized Controlled Trials
ICU: Intensive Care Unit
RRT: Renal Replacement Therapy
SSC: Surviving Sepsis Campaign
TTE: Transthoracic Echocardiogram
IVC: Inferior Vena Cava
LVOT: Left Ventricular Outflow Tract
VTI: Velocity Time Integral
PLR: Passive Leg Raising
SV: Stroke Volume
PPV: Pulse Pressure Variation
CO: Cardiac Output
IVC-CI: Inferior Vena Cava Collapsibility Index
