# Supplementary material for: Personalized fluid resuscitation may reduce mortality in septic shock as compared with fixed-volume approach – a systematic review and meta-analysis of randomized controlled trials
Source: Intensive Care Med Exp. 2026 Sep 28;14:126. doi: 10.1186/s40635-026-00967-5 (PMC13620029; doi:10.1186/s40635-026-00967-5)
Supplement: Supplementary file 1 [file 40635_2026_967_MOESM1_ESM.docx]

**SUPPLEMENTARY MATERIAL**

**Personalized Fluid Resuscitation May Reduce Mortality in Septic Shock as Compared to Fixed-Volume Approach– A Systematic Review and Meta-Analysis of Randomized Controlled Trials**

**Authors**

Gábor Nagy^1^, Júlia Hollósi^1^, Caner Turan^1,4,5,6^, Dávid Laczkó^1,5^, Zoltán Sipos^1^, Péter Hegyi^1,2,3^, László Zubek^1,4^, Zsolt Molnár^1,4,6^

**Affiliations:**

1. Centre for Translational Medicine, Semmelweis University, Budapest, Hungary

2. Institute for Translational Medicine, Medical School, University of Pécs, Pécs, Hungary

3. Institute of Pancreatic Diseases, Semmelweis University, Budapest, Hungary

4. Department of Anesthesiology and Intensive Therapy, Semmelweis University, Budapest, Hungary

5. Department of Interventional Radiology, Heart and Vascular Centre, Semmelweis University, Budapest, Hungary

6. Department of Anesthesiology and Intensive Therapy, Poznan University for Medical Sciences, Poznan, Poland

**Corresponding author**

Zsolt Molnár MD, PhD

E-mail address: molnar.zsolt1@semmelweis.hu

**TABLES**

**Table 1.:** Geographical and institutional scopre of the included studies

| **Author and publication date** | **Study period** | **Country** | **Number of centers** |
| --- | --- | --- | --- |
|  |  |  |  |
| Afandy et al., 2020 (21) | 2019 June – 2020 January | Egypt | 1 |
| Alhabashy et al., 2021 (22) | 2015 March – 2016 May | Egypt | 1 |
| Chen et al., 2015 (23) | 2014 January – 2014 December | United States | 1 |
| Douglas et al., 2020 (24) | 2016 October – 2019 February | United States and United Kingdom | 13 |
| Elsayed et al., 2021 (25) | N/A | Egypt | 1 |
| Ismail et al., 2019 (26) | 2016 July – 2017 Match | Egypt | 1 |
| Kuan et al., 2016 (27) | 2012 February – 2013 November | Singapore | 1 |
| Lanspa et al., 2018 (28) | 2015 January – 2017 May | United States | 1 |
| Li et al., 2021 (29) | 2044 September – 2016 January | China | 1 |
| Musikatavorn et al., 2021 (30) | 2017 January – 2020 January | Thailand | 1 |

**Table 2.** Baseline clinical characteristics of patients

| **Author and publication date** | **SOFA score** | **APACHE II** | **Vasopressor dose** | **Lactate level** |
| --- | --- | --- | --- | --- |
| Afandy et al., 2020 (21) | not reported | 25.6±3.5 vs. 25.4±3.7 vs. 25.5±3.7 | 0.216±0.097 vs. 0.203±0.099 vs. 0.157±0.122 | 3.0±0,7 vs. 2.7± 0.5 vs. 2.7±0.5 |
| Alhabashy et al., 2021 (22) |  | 23.95±3.45 vs. 24.76±4.26 | 0.3 (0.2-0.34) vs. 0.1 (0.05-0.2) | 6.38±1.62 vs. 6.02±1.51 |
| Chen et al., 2015 (23) | 13 (11-15) vs. 13 (11-15) | 25 (22-28.5) vs. 26 (20.5-30) | 18 (9-35) vs. 13 (7-25)^b^ |  |
| Douglas et al., 2020 (24) |  |  |  | 3.6±3.2 vs. 3.8±6.3 |
| Elsayed et al., 2021 (25) | 10.16±2.5 vs. 10.6±2.27 | 26.24±6.02 vs. 24±5.81 |  | 5.8 (2.8-14.2) vs. 5.88 (2.9-14.9) |
| Ismail et al., 2019 (26) | 8 vs. 9^a^ | 24 vs. 26^a^ |  |  |
| Kuan et al., 2016 (27) | 3.6±3 vs. 3.2±2.1 |  |  | 4.8±2 vs. 4.8±2 |
| Lanspa et al., 2018 (28) |  | 29 (23-41) vs. 33 (31-41) |  | 3.6 (2.3-5.0) vs. 3.6 (2.0-5.9) |
| Li et al., 2021 (29) | 13.2±4.8 vs. 13.2±4.1 | 20.8±8.2 vs. 21.8±6.7 | 0.75 (0.2-2.0) vs. 1 (0.35-2.0)^b^ | 4.2 (2.3-6.9) vs. 4.0 (2.6-5.6) |
| Musikatavorn et al., 2021 (30) | 4 (3-6) vs. 4 (3-6) |  |  | 3.2 (2.3-4.1) vs. 3.6 (2.4-5.6) |

Data are presented as mean ± SD, median (IQR) or number (%)
a: no measure of spread was given
b: maximum dose of vasopressor
SOFA: Sequential Organ Failure Assessment, APACHE: Acute Physiology and Chronic Health Evaluation

**Table 3.** Etiology and source of infection

| **Author and publication date** | **Source of infection** |
| --- | --- |
| Afandy et al., 2020 (21) | abscess: 6(20) vs. 3(10) vs. 5(16.7) bronchiectasis: 5(16.7) vs. 5(16.7) vs. 4(13.3) endocarditis: 5(16.7) vs. 6(20%) vs. 4(13.3) peritonitis: 7(23.3) vs. 7(23.3%) vs. 7(23.3) pneumonia: (7(23.3) vs. 9(30) vs. 10(33.3) |
| Alhabashy et al., 2021 (22) | respiratory tract: 16(38.1) vs. 19(42.2) soft tissue: 7(16.7) vs. 11(24.4) bloodstream: 6(14.3) vs. 5(11.1) urinary tract: 6(14.3) vs. 4(8.9) bdomen: 5(11.9) vs. 4(8.9) endocarditis: 1(2.4) vs. 0(0) unknown: 1(2.4) vs. 2 (4.4) |
| Chen et al., 2015 (23) | not reporetd |
| Douglas et al., 2020 (24) | bacterial: 78(76.5) vs. 38(79.2) viral: 6(5.9) vs. 3(6.3) fungal: 1(1) vs. 1(2.1) other: 16(15.7) vs. 6(12.5) unknown: 1(1) vs. 0(0) |
| Elsayed et al., 2021 (25) | respiratory: 16(32) vs. 18(36) abdominal: 6812) vs. 10(20) urinary tract: 10(20) vs. 8(16) skin, soft tissue: 10(20) vs. 6(12) catheter-related: 4(8) vs. 2(4) unknown: 488) vs. 6(12) |
| Ismail et al., 2019 (26) | pneumonia: 21(52.5) vs. 19(47.5) |
| Kuan et al., 2016 (27) | not reported |
| Lanspa et al., 2018 (28) | abdominal: 1(6.7) vs. 2 (13.3)  pulmonary: 6(40) vs. 6(40) skin or soft tissue: 2(13.3) vs. 1(6.7) urinary tract: 4(26.7) vs. 3(20) other/unknown: 2(13.3) vs. 3(20) |
| Li et al., 2021 (29) | urinary tract: 4(8.2) vs. 10(22.2) pneumonia: 10(20.4) vs. 6(13.3) bloodstream: 23(46.9) vs. 20(44.4) peritonitis: 20(40.8) vs. 20(44.4) meningitis: 5 (10.2) vs. 2 (4.4) cholangitis: 2(4.1) vs. 2(4.4) |
| Musikatavorn et al., 2021 (30) | not reported |

**FIGURES**

**Figure 1.** PRISMA 2020 Flowchart illustrating the updated systematic search and study selection process

**Figure 2.** Leave-one-out sensitivity analysis for 30-day mortality Each row represents the re-calculated pooled effect estimate and 95% CI after excluding the specified study from the analysis, demonstarting the robustness of primary finding.

**Figure 4D.** Forest plot illustrating mechanical ventilation-free days

**Figure 5A.** Funnel plot for the visual assessment of publication bias regarding the 30-day mortality outcome

**Figure 5B.** Funnel plot for the visual assessment of publication bias regarding the ICU stay

**Figure 5C.** Funnel plot for the visual assessment of publication bias regarding the length of hospital stay outcome

**Figure 5D.** Funnel plot for the visual assessment of publication bias regarding the requirement for renal replacement therapy outcome

**Figure 5E.** Funnel plot for the visual assessment of publication bias regarding the duration of mechanical ventilation outcome

**Figure 5F.** Funnel plot for the visual assessment of publication bias regarding the duration of vasopressor therapy outcome

**Figure 6A:** Leave-one-ot sensitivity analysis for length of ICU stay. Each row represents the re-calculated pooled effect estimate and 95% CI after excluding the specified study from the analysis, demonstarting the robustness of primary finding.

**Figure 6B.** Leave-one-ot sensitivity analysis for length of hospital stay. Each row represents the re-calculated pooled effect estimate and 95% CI after excluding the specified study from the analysis, demonstarting the robustness of primary finding.

**Figure 6C.** Leave-one-ot sensitivity analysis for requirement for renal replacement therapy. Each row represents the re-calculated pooled effect estimate and 95% CI after excluding the specified study from the analysis, demonstarting the robustness of primary finding.

**Figure 6D.** Leave-one-ot sensitivity analysis for duration of mechanical ventilatio. Each row represents the re-calculated pooled effect estimate and 95% CI after excluding the specified study from the analysis, demonstarting the robustness of primary finding.

**Figure 6E.** Leave-one-ot sensitivity analysis for duration of vasopressor therapy. Each row represents the re-calculated pooled effect estimate and 95% CI after excluding the specified study from the analysis, demonstarting the robustness of primary finding.

**Figure 7A.** Influence forest plot for length of ICU stay. The analysis demonstrates the contribution of each included study to the overall statistical heterogeneity (I^2^) through a leave-one-out approach.

**Figure 7B.** Influence forest plot for length of hospital stay. The analysis demonstrates the contribution of each included study to the overall statistical heterogeneity (I^2^) through a leave-one-out approach.

**Figure 7C.** Influence forest plot for requirement for renal replacement therapy. The analysis demonstrates the contribution of each included study to the overall statistical heterogeneity (I^2^) through a leave-one-out approach.

**Figure 7D.** Influence forest plot for duration of mechanical ventilation. The analysis demonstrates the contribution of each included study to the overall statistical heterogeneity (I^2^) through a leave-one-out approach.

**Figure 7E.** Influence forest plot for duration of vasopressor therapy. The analysis demonstrates the contribution of each included study to the overall statistical heterogeneity (I^2^) through a leave-one-out approach.

**Figure 8.** Ring diagram to aid policy makers in decision-making

**PRISMA 2020 Checklist**

| **Section and Topic** | **Item #** | **Checklist item** | **Location where item is reported** |
| --- | --- | --- | --- |
| **TITLE** | | |  |
| Title | 1 | Identify the report as a systematic review. | Page 1 |
| **ABSTRACT** | | |  |
| Abstract | 2 | See the PRISMA 2020 for Abstracts checklist. | Page 3 |
| **INTRODUCTION** | | |  |
| Rationale | 3 | Describe the rationale for the review in the context of existing knowledge. | Page 4 |
| Objectives | 4 | Provide an explicit statement of the objective(s) or question(s) the review addresses. | Page 4 |
| **METHODS** | | |  |
| Eligibility criteria | 5 | Specify the inclusion and exclusion criteria for the review and how studies were grouped for the syntheses. | Page 5 |
| Information sources | 6 | Specify all databases, registers, websites, organisations, reference lists and other sources searched or consulted to identify studies. Specify the date when each source was last searched or consulted. | Page 5 |
| Search strategy | 7 | Present the full search strategies for all databases, registers and websites, including any filters and limits used. | Page 5 |
| Selection process | 8 | Specify the methods used to decide whether a study met the inclusion criteria of the review, including how many reviewers screened each record and each report retrieved, whether they worked independently, and if applicable, details of automation tools used in the process. | Page 6 |
| Data collection process | 9 | Specify the methods used to collect data from reports, including how many reviewers collected data from each report, whether they worked independently, any processes for obtaining or confirming data from study investigators, and if applicable, details of automation tools used in the process. | Page 5-6 |
| Data items | 10a | List and define all outcomes for which data were sought. Specify whether all results that were compatible with each outcome domain in each study were sought (e.g. for all measures, time points, analyses), and if not, the methods used to decide which results to collect. | Page 5-6 |
|  | 10b | List and define all other variables for which data were sought (e.g. participant and intervention characteristics, funding sources). Describe any assumptions made about any missing or unclear information. | - |
| Study risk of bias assessment | 11 | Specify the methods used to assess risk of bias in the included studies, including details of the tool(s) used, how many reviewers assessed each study and whether they worked independently, and if applicable, details of automation tools used in the process. | Page 6 |
| Effect measures | 12 | Specify for each outcome the effect measure(s) (e.g. risk ratio, mean difference) used in the synthesis or presentation of results. | Page 6 |
| Synthesis methods | 13a | Describe the processes used to decide which studies were eligible for each synthesis (e.g. tabulating the study intervention characteristics and comparing against the planned groups for each synthesis (item #5)). | Page 6 |
|  | 13b | Describe any methods required to prepare the data for presentation or synthesis, such as handling of missing summary statistics, or data conversions. | Page 6 |
|  | 13c | Describe any methods used to tabulate or visually display results of individual studies and syntheses. | Page 6 |
|  | 13d | Describe any methods used to synthesize results and provide a rationale for the choice(s). If meta-analysis was performed, describe the model(s), method(s) to identify the presence and extent of statistical heterogeneity, and software package(s) used. | Page 6 |
|  | 13e | Describe any methods used to explore possible causes of heterogeneity among study results (e.g. subgroup analysis, meta-regression). | Page 6 |
|  | 13f | Describe any sensitivity analyses conducted to assess robustness of the synthesized results. | Page 6 |
| Reporting bias assessment | 14 | Describe any methods used to assess risk of bias due to missing results in a synthesis (arising from reporting biases). | - |
| Certainty assessment | 15 | Describe any methods used to assess certainty (or confidence) in the body of evidence for an outcome. | Page 6 |
| **RESULTS** | | |  |
| Study selection | 16a | Describe the results of the search and selection process, from the number of records identified in the search to the number of studies included in the review, ideally using a flow diagram. | Page 12 |
|  | 16b | Cite studies that might appear to meet the inclusion criteria, but which were excluded, and explain why they were excluded. | Page 12 |
| Study characteristics | 17 | Cite each included study and present its characteristics. | Page 16 |
| Risk of bias in studies | 18 | Present assessments of risk of bias for each included study. | Page 17 |
| Results of individual studies | 19 | For all outcomes, present, for each study: (a) summary statistics for each group (where appropriate) and (b) an effect estimate and its precision (e.g. confidence/credible interval), ideally using structured tables or plots. | Page 7-8 |
| Results of syntheses | 20a | For each synthesis, briefly summarise the characteristics and risk of bias among contributing studies. | - |
|  | 20b | Present results of all statistical syntheses conducted. If meta-analysis was done, present for each the summary estimate and its precision (e.g. confidence/credible interval) and measures of statistical heterogeneity. If comparing groups, describe the direction of the effect. | Page 7-8 |
|  | 20c | Present results of all investigations of possible causes of heterogeneity among study results. | Page 7-8 |
|  | 20d | Present results of all sensitivity analyses conducted to assess the robustness of the synthesized results. | - |
| Reporting biases | 21 | Present assessments of risk of bias due to missing results (arising from reporting biases) for each synthesis assessed. | - |
| Certainty of evidence | 22 | Present assessments of certainty (or confidence) in the body of evidence for each outcome assessed. | Page 2 |
| **DISCUSSION** | | |  |
| Discussion | 23a | Provide a general interpretation of the results in the context of other evidence. | Page 9-10 |
|  | 23b | Discuss any limitations of the evidence included in the review. | Page 10 |
|  | 23c | Discuss any limitations of the review processes used. | - |
|  | 23d | Discuss implications of the results for practice, policy, and future research. | Page 10 |
| **OTHER INFORMATION** | | |  |
| Registration and protocol | 24a | Provide registration information for the review, including register name and registration number, or state that the review was not registered. | Page 7 |
|  | 24b | Indicate where the review protocol can be accessed, or state that a protocol was not prepared. | - |
|  | 24c | Describe and explain any amendments to information provided at registration or in the protocol. | - |
| Support | 25 | Describe sources of financial or non-financial support for the review, and the role of the funders or sponsors in the review. | Page 2 |
| Competing interests | 26 | Declare any competing interests of review authors. | Page 2 |
| Availability of data, code and other materials | 27 | Report which of the following are publicly available and where they can be found: template data collection forms; data extracted from included studies; data used for all analyses; analytic code; any other materials used in the review. | References |

*From:* Page MJ, McKenzie JE, Bossuyt PM, Boutron I, Hoffmann TC, Mulrow CD, et al. The PRISMA 2020 statement: an updated guideline for reporting systematic reviews. BMJ 2021;372:n71. doi: 10.1136/bmj.n71. This work is licensed under CC BY 4.0. To view a copy of this license, visit <https://creativecommons.org/licenses/by/4.0/>
